# Transcranial magnetic stimulation-based multisite neuromodulation for motor recovery after spinal cord injury: a structured narrative review

**DOI:** 10.3389/fneur.2026.2000924

**Published:** 2026-10-27

**Authors:** Yuanbiao Luo, Xinwei Huang, Kun Wang, Shunxiu Lu, Jiawen Song, Xin Yang, Feng Li, Ye Luo

**Affiliations:** 1 Department of Orthopaedics, The First Hospital of Putian City, The School of Clinical Medicine, Fujian Medical University, Putian, Fujian, China; 2 Department of Neurology and Neurological Rehabilitation, Yangzhi Rehabilitation Hospital (Shanghai Sunshine Rehabilitation Center), School of Medicine, Tongji University, Shanghai, China; 3 Department of Rehabilitation Therapy, Kunshan Rehabilitation Hospital, Suzhou, Jiangsu, China; 4 Department of Rehabilitation Medicine, Civil Aviation Shanghai Hospital, Gubei Branch of Ruijin Hospital Affiliated to Shanghai Jiao Tong University School of Medicine, Shanghai, China; 5 Department of Rehabilitation Medicine, Xiang'an Hospital of Xiamen University, School of Medicine, Xiamen University, Xiamen, China; 6 School of Exercise and Health, Shanghai University of Sport, Shanghai, China

**Keywords:** corticospinal plasticity, motor recovery, multisite neuromodulation, paired associative stimulation, spinal cord injury, transcranial magnetic stimulation

## Abstract

Motor impairment after spinal cord injury (SCI) is associated with disrupted corticospinal transmission and altered interactions among supraspinal, spinal, and peripheral sensorimotor pathways. Conventional transcranial magnetic stimulation (TMS) generally targets the motor cortex alone, whereas multisite approaches combine TMS with peripheral nerve, spinal cord, or nerve-root stimulation. This structured narrative review searched PubMed, Web of Science Core Collection, and SPORTDiscus from database inception through August 1, 2026. The searches identified 260 records, of which 100 unique database records remained after deduplication; five additional records were identified through citation tracking and targeted author searches. Single case reports, conference abstracts, and trial protocols were excluded from the evidence synthesis. Twenty-six eligible publications reporting completed human studies were included in the core synthesis: 17 investigated cortical–peripheral nerve stimulation, seven investigated cortical–spinal stimulation, and two investigated cortical–nerve-root stimulation. One mixed-modality multisegment Hebbian study was considered separately because of its mixed stimulation design. Studies that specified a proposed site of neural interaction and used physiology-based stimulus timing reported timing-dependent changes in corticospinal or spinal excitability. Repeated interventions were also associated with improvements in selected measures of strength, hand function, or walking. Neurophysiological effects were reported more consistently across studies than sustained clinical improvements. Interpretation was limited by small sample sizes, heterogeneous stimulation parameters, overlapping cohorts, limited use of active single-target controls, and differences in treatment exposure. Some paradigms showed timing- or component-dependent neurophysiological effects, but the available evidence does not establish durable clinical efficacy or the added benefit of a second stimulation site across approaches. Future trials should include active single-target comparators, individualized timing where relevant, matched treatment exposure, prespecified stratification by clinically relevant injury characteristics, and long-term clinically meaningful outcomes.

## 1 Introduction

Spinal cord injury (SCI) causes persistent motor, sensory, and autonomic deficits (1). Even when an injury is clinically classified as complete, some axonal continuity and subclinical supraspinal influence may remain across the lesion (2, 3), whereas preserved descending and ascending pathways are more common after incomplete SCI (1). Motor recovery therefore depends not only on increasing cortical excitability, but also on improving transmission through residual corticospinal pathways and restoring effective communication between supraspinal, spinal, and peripheral motor systems (4–6). Transcranial magnetic stimulation (TMS) provides a non-invasive means of modulating the motor cortex and corticospinal system (7, 8), and high-frequency repetitive TMS and intermittent theta-burst stimulation (iTBS) can increase corticospinal excitability (9, 10). In people with SCI, however, the translation of cortical stimulation into motor output depends on spared descending pathways, the excitability of spinal circuits below the lesion, lower motor neuron integrity, and the activity of the targeted motor system (5, 6, 11–13). Accordingly, increasing cortical excitability alone may not lead to meaningful motor improvement when transmission across the lesion or recruitment of spinal and peripheral motor pathways remains limited.

Multisite neuromodulation aims to address this limitation by stimulating two or more spatially distinct neural sites so that descending, ascending, or antidromic volleys can interact within a shared neural pathway or circuit (14–17). Completed human studies have mainly used three anatomical approaches: cortical–peripheral nerve stimulation (5, 11–13, 18–30), cortical–spinal stimulation (31–37), and cortical–nerve-root magnetic stimulation (38, 39). These approaches differ substantially in their temporal organization. Cortical–nerve-root studies have used sequential stimulation blocks separated by approximately 10 min (38, 39), whereas several cortical–spinal studies have delivered cortical and spinal stimulation within the same treatment period (32, 33, 36). In contrast, paired corticospinal-motoneuronal stimulation (PCMS) and timing-dependent brain–spinal paradigms pair stimuli at the millisecond scale according to individual conduction latencies or predefined stimulus order (13, 20, 26, 31, 34, 35, 37). Thus, although these interventions all involve more than one stimulation site, they differ in their neural targets, timing strategies, and proposed sites of interaction.

Previous reviews have examined conventional repetitive TMS, cortical–peripheral paired associative stimulation (PAS), transcutaneous spinal stimulation, or broader multi-target approaches after SCI. Reviews of repetitive TMS have mainly focused on single-site cortical stimulation, whereas the available PAS review has largely focused on TMS combined with high-frequency peripheral nerve stimulation. Reviews of transcutaneous spinal stimulation have primarily addressed spinal and corticospinal excitability, while broader multi-target reviews have included heterogeneous approaches such as electrical stimulation, magnetic stimulation, brain–computer interfaces, and regenerative interventions (14–17, 40). A focused synthesis of TMS-based multisite neuromodulation across cortical–peripheral, cortical–spinal, and cortical–nerve-root approaches is therefore still needed. Accordingly, this structured narrative review examines whether the available human evidence supports an added motor or neurophysiological benefit of TMS-based multisite neuromodulation after SCI. We specifically compare how stimulation timing and the proposed site of neural interaction are defined across paradigms, whether study designs allow the contribution of the second stimulation site to be distinguished, and whether neurophysiological changes are accompanied by sustained improvements in motor function.

## 2 Methods

### 2.1 Review design and search strategy

This study was conducted as a structured narrative review. PubMed, Web of Science Core Collection, and SPORTDiscus were searched from database inception through August 1, 2026; SPORTDiscus was accessed through EBSCOhost. The search strategy combined three concept groups covering SCI, TMS, and paired or multisite stimulation. The same general search concepts and Boolean logic were applied across databases, with field syntax adapted to each platform. Search terms included combinations of “spinal cord injury,” “tetraplegia,” “transcranial magnetic stimulation,” “paired associative stimulation,” “paired corticospinal-motoneuronal stimulation,” “Hebbian stimulation,” “transspinal stimulation,” “peripheral nerve stimulation,” and “nerve root stimulation.” Searches were restricted to title and abstract fields, and no date, language, or publication-type filters were applied. The complete database-specific search strategies, including search terms, Boolean operators, and field tags, are provided in Supplementary Table S1. The searches identified 90 records in PubMed, 77 in Web of Science Core Collection, and 93 in SPORTDiscus, yielding 260 database records in total. Additional records were identified by screening the reference lists of included studies and relevant reviews, forward citation tracking, and targeted author searches. Database records were deduplicated using DOI, PMID, and normalized titles. When a preprint and a peer-reviewed journal article reported the same study, the journal article was retained. Conference abstracts describing an otherwise available study were not treated as separate evidence.

### 2.2 Eligibility criteria

Original human studies of SCI were eligible if:

(1) TMS was used as a therapeutic or mechanistic neuromodulation intervention rather than solely as an assessment tool;

(2) the intervention involved at least two spatially distinct neural stimulation sites;

(3) motor function, strength, walking, hand function, motor control, or motor neurophysiology was assessed; and

(4) original study results were reported. Eligible designs included single-session mechanistic studies, repeated-intervention studies, case series, non-randomized studies, and randomized controlled trials. Studies were excluded if they were single case reports, conference abstracts, trial protocols, studies restricted to healthy participants, conventional single-target repetitive TMS studies, TMS combined with rehabilitation without a second neural stimulation target, studies in which TMS was used only for outcome assessment, spinal or peripheral stimulation without TMS as an intervention component, animal studies, or studies focused exclusively on pain, mood, cognition, autonomic function, or bladder outcomes. Trial protocols were not included in the core study set or outcome synthesis but were considered separately in the future-directions section.

### 2.3 Data extraction and narrative synthesis

Extracted information included study design; participant and SCI characteristics; TMS coil and cortical target; modality and location of the second stimulus; stimulus order and interstimulus interval; stimulation dose and treatment course; concurrent rehabilitation; control and blinding procedures; neurophysiological, motor, and functional outcomes; follow-up; and adverse events. One author performed study selection and data extraction, which were subsequently reviewed by a second author. The distribution of included studies across stimulation routes is summarized in Table 1, and study-level characteristics, stimulation protocols, and reported outcomes are presented in Table 2. Studies were grouped into three main categories: cortical–peripheral nerve stimulation, cortical–spinal stimulation, and cortical–nerve-root stimulation. The 2023 study by Jo and colleagues was considered separately because its upper-limb phase included TMS, whereas its randomized lower-limb phase used thoracic spinal electrical stimulation (41). The Pulverenti and Helsinki high-PAS series included multiple publications from the same projects or overlapping participant cohorts. Eligible publications were retained when they reported distinct mechanistic or clinical outcomes, but publications from overlapping cohorts were not treated as independent patient samples. This included secondary mechanistic reports such as the study by Vanhanen and colleagues. The narrative synthesis compared stimulation targets, timing, dose, control conditions, and whether neurophysiological changes were accompanied by changes in motor or functional outcomes.

**Table 1:** Distribution of completed human publications by stimulation route.

Stimulation route	Publications	Main approach	Main outcomes	Key design features
Cortical–peripheral nerve	17	Cortical PAS, spinal PCMS, high-PAS	MEPs, strength, hand function, walking	Includes single-session mechanistic studies and repeated-treatment studies; several high-PAS publications arose from overlapping cohorts
Cortical–spinal	7	rTMS/iTBS combined with transspinal stimulation; timing-dependent brain–spinal pairing	MEPs, H-reflex, flexion reflex, transspinal evoked potentials, motor scores	Three Pulverenti publications arose from the same project and overlapping participant cohorts
Cortical–nerve-root	2	Sequential cortical iTBS and nerve-root magnetic stimulation	MEPs, LEMS, SCIM, gait	Both studies used sequential cortical and nerve-root stimulation; active single-target comparator groups were not included
Mixed multisegment	1	Upper-limb TMS plus PNS; lower-limb spinal electrical stimulation plus PNS	Strength, grasp, walking, quality of life	Upper- and lower-limb phases used different stimulation modalities and were considered separately from the three main categories

Publication counts do not represent independent projects or pooled participant samples. The mixed-modality study was considered separately and was not included among the 26 core publications. Single case reports, conference abstracts, and trial protocols were excluded from the core synthesis.

**Table 2:** Study characteristics, stimulation protocols, and reported outcomes of 26 core publications.

Stimulation route	Study	Participants and design	Stimulation protocol	Timing and treatment course	Outcomes and main reported findings
Cortical–peripheral nerve stimulation	Roy et al. (11)	13 individuals with incomplete SCI in the PAS experiment; single-session mechanistic study	Cortical PAS: leg M1 TMS + common peroneal nerve PNS	Peripheral input timed to interact with cortical activation; single session	TA MEP; 7/13 showed >20% transient MEP facilitation after PAS
	Bunday and Perez (12)	19 individuals with chronic cervical SCI; within-subject mechanistic study	PCMS: hand M1 TMS + ulnar nerve PNS	Individualized conduction-based timing; descending volley arrived ~1–2 ms before the antidromic volley; single session	MEP, finger force, Nine-Hole Peg Test; facilitatory timing increased corticospinal transmission, force, and hand performance
	Urbin et al. (13)	18 individuals with chronic incomplete SCI; lower-limb study	PCMS: leg M1 TMS + common peroneal nerve PNS	Individualized millisecond-level timing; single session	TA MEP, EMG, dorsiflexion force; short-term increases were reported after facilitatory pairing
	Bunday et al. (20)	17 individuals with chronic SCI; within-subject mechanistic study	PCMS: M1 TMS + peripheral motor-nerve stimulation	Conduction-based pairing tested at rest and during mild voluntary contraction	MEP-related measures; mild voluntary contraction increased the PCMS response
	Donges et al. (22)	11 individuals with chronic cervical SCI; experimental study	PCMS targeting elbow-flexor pathways	Individualized paired stimulation; single-session protocol	Maximal voluntary elbow flexion; no clear improvement was observed
	Jo and Perez (5)	38 individuals with chronic SCI; controlled repeated-treatment study	PCMS: M1 TMS + corresponding peripheral motor-nerve stimulation	Individualized pairing; repeated PCMS with or without exercise	MEP, MVC, functional tasks; MEP and MVC increased in real-PCMS groups, with some changes maintained during follow-up
	Grover et al. (26)	22 individuals with SCI; within-subject dose-comparison study	PCMS: hand M1 TMS + peripheral motor-nerve stimulation	180 vs. 360 paired stimuli	MEP and Nine-Hole Peg Test; 360 pairs produced larger physiological and hand-performance changes than 180 pairs
	Versace et al. (21)	10 individuals with chronic traumatic SCI; pilot study with healthy controls	Conventional cortical PAS	Millisecond-level peripheral–cortical pairing; single session	MEP; PAS responses varied according to residual motor recovery
	Shulga et al. (18)	2 individuals with chronic motor-incomplete SCI; proof-of-concept case series	High-PAS: high-intensity M1 TMS + high-frequency PNS	Individualized latency-based pairing; 20–24-week treatment	MEP and voluntary motor control; voluntary activation returned in selected previously paralyzed muscles
	Tolmacheva et al. (19)	5 individuals with chronic tetraplegia; within-participant controlled study	High-PAS to one hand; PNS + sham TMS to the contralateral hand	Individualized latency-based pairing; repeated treatment	Upper-limb motor output; greater changes were reported in the PAS-treated hand
	Tolmacheva et al. (23)	5 individuals with chronic non-traumatic SCI; case series	Long-term high-PAS targeting upper-limb nerves	Individualized latency-based pairing; repeated multiweek treatment	Hand strength and function; improvements were reported in selected upper-limb measures
	Rodionov et al. (24)	5 individuals with chronic incomplete tetraplegia; proof-of-concept pilot study	Long-term high-PAS targeting lower-limb pathways	Individualized pairing; repeated treatment over several weeks	Leg strength and walking; improvements were maintained at follow-up, whereas AIS sensory score, MAS, and SCIM did not change significantly
	Vanhanen et al. (25)	5 individuals with chronic incomplete SCI; longitudinal mechanistic report from a high-PAS cohort	Long-term high-PAS	Repeated multiweek treatment	Cortical sensorimotor oscillations; treatment was associated with changes in sensorimotor oscillatory activity
	Holopainen et al. (27)	9 individuals with SCI; randomized single-session crossover study	High-PAS with or without music	Individualized PAS delivered under different music conditions	MEP and alertness; music did not further increase PAS-related MEP facilitation
	Wahlgren et al. (28)	5 individuals with chronic incomplete cervical SCI; prospective study	Long-term high-PAS targeting one upper limb	Repeated treatment over 4 weeks	Upper-limb strength and self-rated performance; improvements were reported after treatment
	Holopainen et al. (29)	4 individuals with incomplete cervical SCI; long-term case series	High-PAS targeting upper-limb peripheral nerves	Individualized repeated pairing over several weeks	Hand muscle strength; improvements were reported and remained stable during follow-up
	Shulga et al. (30)	17 individuals with subacute incomplete cervical SCI; randomized double-blind sham-controlled study	Repeated high-PAS	Active or sham PAS during the subacute rehabilitation period	Muscle strength and fine hand function; greater improvements were reported for selected weaker muscles and fine hand outcomes
Cortical–spinal stimulation	Zhang et al. (32)	3 individuals with incomplete SCI; preliminary pilot study	rTMS + transspinal direct-current stimulation	Different cortical–spinal waveform combinations tested	Corticospinal excitability; combined 20-Hz rTMS + cathodal tsDCS produced larger changes than single-target conditions
	Pulverenti et al. (31)	14 individuals with chronic SCI; randomized clinical study	M1 TMS + thoracolumbar transcutaneous spinal stimulation	TMS-before-spinal or spinal-before-TMS during stance; 240 paired stimuli/session with locomotor training	H-reflex and locomotor EMG; stimulation order produced different phase-dependent responses
	Pulverenti et al. (34)	Overlapping cohort from the same project; secondary physiological report	M1 TMS + thoracolumbar transspinal stimulation	Timing-dependent pairing during robot-assisted walking	Flexion reflex; stimulation order differentially modulated polysynaptic reflex circuits
	Pulverenti et al. (35)	10 individuals with chronic SCI from the same project; trial report	M1 TMS + thoracolumbar transspinal stimulation	240 pairs/session; ≥20 sessions during locomotor training	Transspinal-evoked potentials and motor output; knee and ankle responses showed phase- and order-dependent changes
	Adeel et al. (33)	9 individuals with chronic incomplete SCI; randomized-order crossover study	20-Hz rTMS + tsDCS or iTBS + tsDCS	Acute combined stimulation followed by bicycling	MEP, H-reflex, LEMS, MAS; corticospinal measures changed after paired stimulation, and LEMS increased after iTBS/tsDCS
	Lin et al. (36)	12 individuals with incomplete SCI; randomized four-group pilot study	iTBS + tsDCS, iTBS alone, tsDCS alone, or sham	8-week treatment + 4-week treatment-free observation	MEP and clinical motor measures; combined stimulation produced a larger MEP increase, which declined after treatment ended
	Murray et al. (37)	16 individuals with chronic cervical SCI; acute mechanistic study	Motor-cortex TMS + cervical transcutaneous spinal stimulation	Pairing intervals systematically varied from −50 to +30 ms	Upper-limb MEP; facilitation occurred when cortical and spinal inputs converged within an appropriate timing window
Cortical–nerve-root stimulation	Mao et al. (38)	14 individuals with chronic SCI and lower-limb dysfunction; prospective case series	M1 iTBS + bilateral lumbar nerve-root magnetic stimulation	Root stimulation ~10 min after cortical iTBS; 5 sessions/week for 4 weeks + rehabilitation	MEP, LEMS, balance, SCIM, walking; neurophysiological and motor-function measures improved after treatment
	Deng et al. (39)	30 individuals with incomplete SCI and lower-limb dysfunction; randomized single-blind sham-controlled trial	Cortical iTBS + magnetic stimulation of nerve roots at the injury level	Sequential dual-target treatment for 21 days + conventional rehabilitation	MEP, knee strength, LEMS, SCIM, gait; active treatment showed greater improvements in several motor and functional outcomes than sham

## 3 Human evidence and study characteristics

### 3.1 Study selection and overall characteristics

The three database searches yielded 260 records: 90 from PubMed, 77 from Web of Science Core Collection, and 93 from SPORTDiscus. After removal of 160 duplicates based on DOI, PMID, and normalized title, 100 unique database records remained. Reference-list screening, forward citation tracking, and targeted author searches identified five additional unique records, resulting in 105 records assessed against the eligibility criteria. Of these, 78 were excluded. Twenty-six eligible publications reporting completed human studies formed the core set: 17 investigated cortical–peripheral nerve stimulation (5, 11–13, 18–30), seven investigated cortical–spinal stimulation (31–37), and two investigated cortical–nerve-root stimulation (38, 39). One mixed-modality multisegment Hebbian study was considered separately because its upper- and lower-limb phases used different stimulation approaches (41). Single case reports, conference abstracts, and trial protocols were not included in the core set. Sample sizes were generally small, and the included studies involved individuals with subacute or chronic, traumatic or non-traumatic SCI across cervical, thoracic, and thoracolumbar injury levels. Study designs ranged from single-session mechanistic experiments and case series to sham-controlled trials and studies with active comparison conditions. Several publications arose from the same project or overlapping participant cohorts; therefore, participant numbers were not pooled across reports. The study-selection process is summarized in Figure 1, the distribution of studies by stimulation route is presented in Table 1, and study characteristics, stimulation protocols, and reported outcomes are summarized in Table 2.

**Figure 1:**
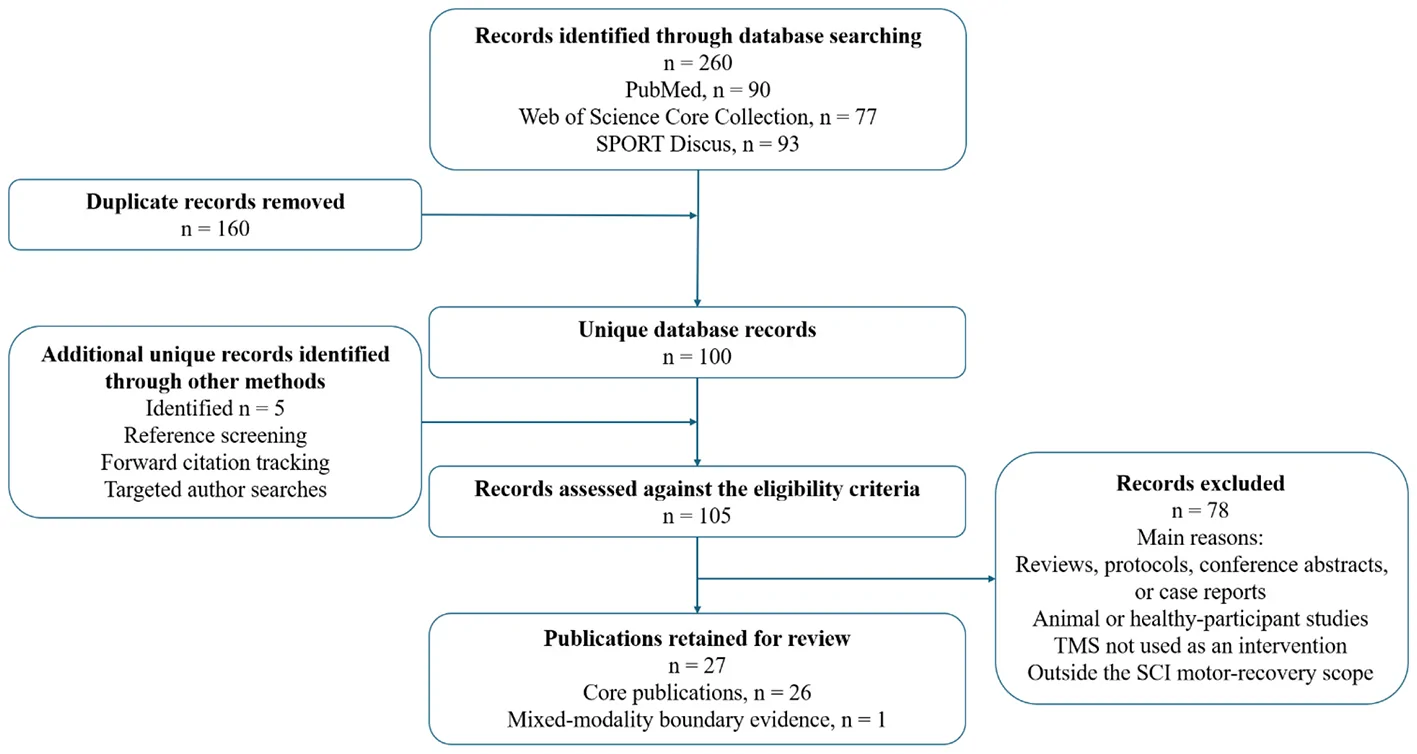
Combined literature identification and eligibility summary. Database searches identified 260 records, of which 160 duplicates were removed, leaving 100 unique records. Five additional records were identified through reference-list screening, forward citation tracking, and targeted author searches, resulting in 105 records assessed for eligibility. Of these, 78 were excluded, 26 eligible publications reporting completed human studies were included in the core synthesis, and one mixed-modality study was considered separately.

### 3.2 Cortical-peripheral nerve stimulation

#### 3.2.1 Cortical PAS

Conventional cortical paired associative stimulation (PAS) pairs peripheral afferent stimulation with motor-cortex TMS, with stimulus timing selected to influence cortical excitability. Roy and colleagues reported a transient increase in tibialis anterior motor-evoked potentials (MEPs) in a subset of participants with incomplete SCI, whereas Versace and colleagues found variable PAS responses that differed according to residual sensorimotor function and previous motor recovery (11, 21). These studies indicate that peripheral afferent stimulation can modulate corticospinal excitability after SCI, although the reported effects were mainly short-term and neurophysiological.

#### 3.2.2 Spinal PCMS

Paired corticospinal-motoneuronal stimulation (PCMS) combines a descending corticospinal volley induced by TMS with an antidromic volley induced by peripheral motor-nerve stimulation. The stimuli are timed to interact at the corticospinal-motoneuronal synapse in the spinal cord. Bunday and Perez individualized the interstimulus interval so that the descending volley reached the target synapse approximately 1–2 ms before the antidromic volley. This stimulus order increased MEPs, finger force, and Nine-Hole Peg Test performance, whereas the reverse order did not produce the same changes (12). Urbin and colleagues extended the approach to the lower limb and reported short-term increases in tibialis anterior MEPs, electromyographic activity, and dorsiflexion force (13).

Subsequent studies examined the effects of voluntary activation and stimulation dose. Mild voluntary contraction increased the PCMS response (20), and Grover and colleagues compared different numbers of stimulus pairs, reporting larger neurophysiological and hand-function changes with the higher number of pairs (26). In contrast, Dongés and colleagues did not find a clear improvement in maximal voluntary elbow flexion (22), indicating that changes in neurophysiological measures were not consistently accompanied by improvement across motor tasks.

Jo and Perez subsequently examined repeated PCMS with and without exercise. Thirty-eight people with chronic SCI received PCMS plus exercise, sham PCMS plus exercise, or PCMS alone. MEPs and maximal voluntary contraction increased in the two active-PCMS groups, whereas short-term functional task performance improved across groups. In participants who completed follow-up, some changes were maintained for up to 6 months in the PCMS-plus-exercise group (5). These findings extend PCMS from single-session experiments to repeated treatment, but the additional functional effect of combining PCMS with exercise was not consistent across outcomes.

#### 3.2.3 High-PAS

High-PAS combines high-intensity TMS with high-frequency peripheral nerve stimulation and has been examined in both single-session experiments and repeated-treatment protocols. Shulga and colleagues treated two people with chronic motor-incomplete SCI for 20–24 weeks and reported recovery of voluntary control in muscles that had previously been paralyzed (18). Tolmacheva and colleagues applied PAS to one hand and peripheral nerve stimulation with sham TMS to the contralateral hand, allowing the contribution of the TMS component to be examined within the same individuals (19). Subsequent studies extended high-PAS to non-traumatic SCI, lower-limb strength and walking, and shorter upper-limb treatment courses (23, 24, 28, 29). These studies generally reported improvements in selected measures of muscle strength or upper- and lower-limb function.

A randomized, double-blind, sham-controlled study published in 2026 evaluated repeated high-PAS during the subacute stage after cervical SCI. Greater improvements were reported in selected weak muscles and fine motor performance in the active group, whereas broader measures of independence did not differ clearly between groups (30). This study extended previous case-series findings to a sham-controlled design and longer follow-up, while the observed benefits remained concentrated in selected motor outcomes.

### 3.3 Cortical-spinal stimulation

#### 3.3.1 Concurrent cortical and transspinal stimulation

Zhang and colleagues developed a system combining TMS with transspinal direct-current stimulation and compared different stimulation combinations in a small sample of individuals with incomplete SCI (32). Adeel and colleagues subsequently examined 20-Hz rTMS or iTBS combined with transspinal direct-current stimulation and reported changes in corticospinal excitability and selected lower-limb motor outcomes (33). Lin and colleagues compared combined iTBS and transspinal stimulation with iTBS alone, transspinal stimulation alone, and sham stimulation. The combined group showed a larger increase in MEP amplitude, although this effect decreased after treatment and was not accompanied by an equally clear sustained difference in clinical motor outcomes (36). Together, these studies indicate that cortical and spinal stimulation can be delivered within the same treatment session, but the added benefit of combining the two sites was not consistent across neurophysiological and clinical outcomes.

#### 3.3.2 Timing-dependent brain-spinal pairing

The Pulverenti group paired single-pulse TMS with thoracolumbar transcutaneous spinal stimulation during the stance phase of robot-assisted walking. Three publications from the same registered project reported effects on soleus H-reflexes, tibialis anterior flexion reflexes, and multimuscle transspinal evoked potentials and represented different outcomes from overlapping participant cohorts rather than independent samples (31, 34, 35). Different stimulus orders were associated with different changes in spinal reflexes and motor output, indicating that the temporal order of cortical and spinal stimulation influenced the response. Because the intervention was delivered together with locomotor training and did not include separate cortical-only or spinal-only treatment groups, the contribution of paired stimulation could not be isolated from the accompanying training.

Murray and colleagues systematically varied the interval between motor-cortex TMS and cervical transcutaneous spinal stimulation in people with chronic cervical SCI. Acute facilitation of upper-limb motor responses occurred within a specific timing window, whereas other intervals produced smaller changes (37). This study provided direct information on the timing dependence of brain–spinal stimulation and identified intervals that could be examined in repeated-treatment studies.

### 3.4 Cortical-nerve-root magnetic stimulation

Mao and colleagues delivered bilateral lumbar nerve-root magnetic stimulation approximately 10 min after motor-cortex iTBS. MEPs, lower-extremity motor scores, balance, independence, and walking measures improved after treatment (38). Because the study used a pre–post design without a separate control group, the contribution of the combined stimulation could not be distinguished from concurrent rehabilitation or changes over time.

Deng and colleagues subsequently conducted a randomized, single-blind, sham-controlled study combining cortical iTBS with magnetic stimulation of nerve roots at the injury level. Compared with the sham group, the active group showed greater improvements in MEPs, knee flexion and extension strength, lower-extremity motor scores, independence, and selected gait measures (39). However, cortical-only and nerve-root-only treatment groups were not included, so the specific contribution of each stimulation component was not examined. Unlike PCMS, both cortical–nerve-root studies used sequential stimulation separated by minutes rather than millisecond-scale timing based on neural conduction.

### 3.5 Mixed multimuscle and multisegment Hebbian stimulation

Jo and colleagues examined a multimuscle and multisegment Hebbian stimulation approach in people with chronic SCI (41). In the randomized lower-limb phase, participants received active or sham stimulation combined with exercise, with the active intervention using thoracic spinal electrical stimulation paired with peripheral nerve stimulation. This phase therefore did not include therapeutic TMS. In a subsequent prospective phase, bilateral M1 TMS was paired with brachial plexus or ulnar nerve stimulation to target upper-limb muscles. Across the study phases, improvements were reported in measures of strength, grasp, walking, and selected quality-of-life outcomes (41).

### 3.6 Safety, tolerability, and follow-up

Across studies that reported adverse events, no serious stimulation-related adverse events were reported. Tolerability issues in long-term high-PAS studies mainly involved transient discomfort associated with peripheral nerve stimulation and the burden of prolonged treatment sessions. Some protocols gradually increased peripheral stimulation intensity during the first sessions, and topical lidocaine/prilocaine cream was used in selected studies to reduce local discomfort. Difficulty remaining seated during long stimulation sessions was also reported by some participants (19, 29, 30, 42). A separate SCI study examining heart-rate variability during a single high-PAS session found no sustained increase in sympathetic activity during or after stimulation (42). Follow-up periods ranged from several weeks to 6–12 months in several repeated-treatment studies, although the number of participants available at follow-up was often smaller than at baseline. Longer-term safety across different TMS intensities, peripheral stimulation doses, spinal stimulation methods, and stages of SCI has not yet been systematically compared.

## 4 Discussion

### 4.1 Mechanistic rationale for multisite neuromodulation

Multisite stimulation should be interpreted according to both the neural sites stimulated and the timing between stimuli. For example, M1 TMS and ulnar nerve stimulation are delivered at the scalp and wrist, but in PCMS the stimuli are timed to interact at corticospinal-motoneuronal synapses in the spinal cord. By contrast, nerve-root stimulation delivered several minutes after motor-cortex iTBS does not involve millisecond-scale pairing and may act through more prolonged changes in neural excitability. The use of two stimulation sites alone therefore does not establish an associative interaction between the two stimuli.

The rationale for precisely timed pairing is largely based on Hebbian and spike-timing-dependent mechanisms. Repeated pairing may produce different neurophysiological effects depending on the order and relative arrival time of the two inputs. Studies of spinal associative stimulation and related paired-stimulation paradigms in neurologically intact participants have shown that changing the interstimulus interval can alter the magnitude and direction of the response (43–45). Similar timing-dependent effects were reported after SCI in the studies by Bunday, Urbin, Pulverenti, and Murray, in which stimulus order or estimated arrival time influenced corticospinal or spinal responses (12, 13, 31, 34, 35, 37). These findings indicate that stimulation timing and the proposed site of neural interaction are important when comparing multisite approaches, rather than simply the number of stimulation sites or pulses delivered.

### 4.2 Timing, dose, and task state

The included studies used markedly different temporal arrangements, ranging from sequential interventions separated by minutes to concurrent stimulation within the same treatment session and individualized pairing at the millisecond scale. These approaches are unlikely to act through the same mechanism and should not be considered equivalent forms of PAS. Studies of high-PAS have also shown that stimulation frequency, pulse configuration, and intensity can influence the magnitude and duration of MEP changes (46, 47). Future studies should therefore report stimulus order, interstimulus interval, how the interval was determined, and whether timing was individualized according to conduction latency. When an associative effect is proposed, reverse timing, non-convergent timing, or active single-target conditions can help determine whether the response depends on the interaction between the two stimuli.

Stimulation dose also varied substantially. Single-session studies generally used approximately 100–360 paired stimuli, whereas repeated-treatment studies ranged from 10–40 sessions, with some high-PAS protocols extending beyond 20 weeks. Grover and colleagues directly compared different numbers of stimulus pairs and reported larger responses with the higher number of pairs within the range tested (26). Across studies, however, longer treatment courses were also associated with differences in participant characteristics, targeted muscles, concurrent rehabilitation, and outcome measures. Treatment dose should therefore be described separately for each stimulation site, together with total treatment duration and concurrent rehabilitation exposure.

The motor state during or around stimulation may also influence the response. Mild voluntary contraction increased the response to PCMS (20), stimulation delivered during specific phases of walking produced phase-dependent changes in spinal and motor output (31, 35), and Jo and Perez combined repeated PCMS with exercise performed after stimulation (5). These studies indicate that voluntary activation and concurrent motor activity can influence the effects of stimulation. The optimal task, timing of rehabilitation relative to stimulation, and whether different muscles require different timing or dose parameters remain unclear.

### 4.3 Patient heterogeneity and individualized parameters

Neurological level, AIS grade, time since injury, the presence of recordable MEPs, residual sensory conduction, lower motor neuron integrity, and the ability to perform the target motor task may influence responses to multisite stimulation. Upper-limb stimulation after cervical SCI and lower-limb stimulation after thoracic SCI also differ in conduction distance and the neural pathways available below the lesion. In subacute SCI, changes during treatment may additionally be influenced by spontaneous recovery. These characteristics have not been examined consistently across studies because most cohorts were small. Future trials should account for clinically relevant injury characteristics in randomization and prespecify how stimulation targets and timing will be individualized.

### 4.4 From neurophysiology to functional recovery

MEPs, cervicomedullary responses, H-reflexes, flexion reflexes, and transspinal evoked potentials provide information about changes in corticospinal or spinal excitability, but these changes do not necessarily correspond to improvement in motor function. Dongés and colleagues did not observe a clear improvement in maximal voluntary elbow flexion despite the use of PCMS (22). In the study by Lin and colleagues, changes in MEP amplitude were more apparent than sustained differences in clinical motor outcomes (36), while Jo and Perez found that short-term task performance also improved in the exercise control group (5). Conversely, several long-term high-PAS studies reported improvements in strength or function but used case-series designs or limited active controls (18, 23, 24, 28, 29). Across the included studies, changes in neurophysiological measures were reported more consistently than sustained improvements in activity or functional independence. Future trials should include both mechanistic measures and prespecified clinical outcomes and should report the magnitude and durability of functional change.

### 4.5 Added value of the second target

Whether a second stimulation site provides additional benefit depends strongly on the control design. Comparisons between a combined intervention and sham treatment test the combined intervention as a whole, whereas comparisons with an active single-target condition can determine whether adding the second stimulation site changes the outcome. Reverse or non-convergent timing can test whether responses depend on stimulus timing, while comparisons of different pair numbers can examine dose within a given protocol. Similarly, stimulation plus exercise compared with sham stimulation plus the same exercise can determine whether stimulation adds to the effects of training. Examples include the reverse-timing condition used by Bunday and Perez (12), high-PAS applied to one hand compared with peripheral nerve stimulation plus sham TMS in the contralateral hand (19), the component comparisons used by Lin and colleagues (36), and the active- and sham-PCMS exercise groups examined by Jo and Perez (5). These studies show that timing or the presence of an additional stimulation component can affect outcomes in some paradigms, but this has not been established across all multisite approaches. Cortical–nerve-root studies and several cortical–spinal studies reported improvements with combined treatment, but most did not include both cortical-only and second-site-only comparator groups. The added benefit of the second stimulation site therefore remains unclear for several approaches. Future trials should use active single-target comparators when the aim is to determine whether the additional stimulation site justifies the added complexity of treatment.

### 4.6 Clinical feasibility

Clinical implementation also depends on the technical and practical demands of treatment. High-PAS may require sequential stimulation of several peripheral nerves, brain–spinal pairing may require accurate synchronization with stimulus timing or gait phase, and cortical–nerve-root protocols require stimulation at two anatomically distinct sites. These requirements can increase preparation time, treatment duration, and staff training needs. Future studies should therefore report preparation and treatment time, adherence, tolerability, withdrawal reasons, blinding success where relevant, and practical difficulties encountered during treatment. Such information will help determine whether more complex multisite protocols can be implemented reliably alongside routine rehabilitation.

## 5 Limitations and future directions

This review and the included literature have several limitations. As a structured narrative review, it did not include formal risk-of-bias assessment or meta-analysis. Despite second-author verification of study selection and data extraction, potential selection and interpretation bias inherent to narrative synthesis cannot be excluded. The underlying studies were also heterogeneous in participant characteristics, stimulation parameters, treatment duration, concurrent rehabilitation, and outcome measures. Study designs also varied in evidential strength, with mechanistic studies primarily informing short-term neurophysiological effects and uncontrolled studies providing less certain evidence of sustained clinical benefit than controlled trials. Several publications from the Pulverenti and Helsinki high-PAS series were derived from overlapping projects or participant cohorts and therefore should not be interpreted as independent samples. Small sample sizes and limited use of active comparator groups further restricted comparisons across stimulation approaches. Similar concerns regarding sample size, protocol heterogeneity, and methodological limitations have been noted in previous reviews of PAS and transcutaneous spinal stimulation (14, 15).

Future studies should focus on whether the second stimulation site provides benefit beyond a single-target intervention. Active component controls, such as combined stimulation vs. each target alone and matched sham, would help separate multisite effects from differences in total stimulation dose, treatment duration, rehabilitation exposure, or sensory experience. Mechanistic studies should test stimulus timing and order directly, while clinical trials should prespecify primary functional outcomes, use blinded assessment, include longer follow-up, and account for injury level, chronicity, recordable MEPs, and lower motor neuron integrity. Recent TMS–tSCS trial protocols use tSCS-based active comparator conditions, allowing more direct assessment of the added cortical stimulation component (48, 49).

## 6 Conclusions

TMS-based multisite neuromodulation has been applied through cortical–peripheral nerve, cortical–spinal, and cortical–nerve-root approaches using different stimulation timings and treatment schedules. Human studies have reported changes in corticospinal or spinal excitability, and some repeated-treatment studies have also reported improvements in strength, hand function, or walking. Across studies, neurophysiological changes were reported more consistently than sustained clinical improvements. Active single-target comparators, matched treatment exposure, independent replication, and long-term functional outcomes remain limited, and the added benefit of a second stimulation site has not been established across approaches.
