# Supplementary material for: Transcranial magnetic stimulation-based multisite neuromodulation for motor recovery after spinal cord injury: a structured narrative review
Source: Front Neurol. 2026 Oct 27;17:2000924. doi: 10.3389/fneur.2026.2000924 (PMC13750277; doi:10.3389/fneur.2026.2000924)
Supplement: Supplementary file 1 [file Table_1.docx]

**Supplementary Table S1. Complete Database-Specific Search Strategies for TMS-Based Multisite Neuromodulation After Spinal Cord Injury**

| **Database** | **Search platform** | **Search strategy** | **Records identified** |
| --- | --- | --- | --- |
| PubMed | PubMed | ("spinal cord injur*"[Title/Abstract] OR "spinal cord lesion*"[Title/Abstract] OR tetraplegi*[Title/Abstract] OR quadriplegi*[Title/Abstract] OR paraplegi*[Title/Abstract]) AND ("transcranial magnetic stimul*"[Title/Abstract] OR TMS[Title/Abstract] OR rTMS[Title/Abstract] OR iTBS[Title/Abstract] OR "theta burst stimul*"[Title/Abstract]) AND ("paired associative stimul*"[Title/Abstract] OR "paired associative magnetic stimul*"[Title/Abstract] OR "paired corticospinal-motoneuronal stimul*"[Title/Abstract] OR "paired corticospinal motor neuronal stimul*"[Title/Abstract] OR "paired corticomotoneuronal stimul*"[Title/Abstract] OR "paired stimul*"[Title/Abstract] OR "spinal associative stimul*"[Title/Abstract] OR "Hebbian stimul*"[Title/Abstract] OR "multisite stimul*"[Title/Abstract] OR "multi-site stimul*"[Title/Abstract] OR "dual-site stimul*"[Title/Abstract] OR "dual target stimul*"[Title/Abstract] OR "dual-target stimul*"[Title/Abstract] OR "peripheral nerve stimul*"[Title/Abstract] OR "nerve root stimul*"[Title/Abstract] OR "transspinal stimul*"[Title/Abstract] OR "trans-spinal stimul*"[Title/Abstract] OR "transspinal direct current stimul*"[Title/Abstract] OR "transcutaneous spinal cord stimul*"[Title/Abstract] OR "spinal cord stimul*"[Title/Abstract] OR tsDCS[Title/Abstract] OR tSCS[Title/Abstract]) | 90 |
| Web of Science Core Collection | Web of Science | (TI=("spinal cord injur*" OR "spinal cord lesion*" OR tetraplegi* OR quadriplegi* OR paraplegi*) OR AB=("spinal cord injur*" OR "spinal cord lesion*" OR tetraplegi* OR quadriplegi* OR paraplegi*)) AND (TI=("transcranial magnetic stimul*" OR TMS OR rTMS OR iTBS OR "theta burst stimul*") OR AB=("transcranial magnetic stimul*" OR TMS OR rTMS OR iTBS OR "theta burst stimul*")) AND (TI=("paired associative stimul*" OR "paired associative magnetic stimul*" OR "paired corticospinal-motoneuronal stimul*" OR "paired corticospinal motor neuronal stimul*" OR "paired corticomotoneuronal stimul*" OR "paired stimul*" OR "spinal associative stimul*" OR "Hebbian stimul*" OR "multisite stimul*" OR "multi-site stimul*" OR "dual-site stimul*" OR "dual target stimul*" OR "dual-target stimul*" OR "peripheral nerve stimul*" OR "nerve root stimul*" OR "transspinal stimul*" OR "trans-spinal stimul*" OR "transspinal direct current stimul*" OR "transcutaneous spinal cord stimul*" OR "spinal cord stimul*" OR tsDCS OR tSCS) OR AB=("paired associative stimul*" OR "paired associative magnetic stimul*" OR "paired corticospinal-motoneuronal stimul*" OR "paired corticospinal motor neuronal stimul*" OR "paired corticomotoneuronal stimul*" OR "paired stimul*" OR "spinal associative stimul*" OR "Hebbian stimul*" OR "multisite stimul*" OR "multi-site stimul*" OR "dual-site stimul*" OR "dual target stimul*" OR "dual-target stimul*" OR "peripheral nerve stimul*" OR "nerve root stimul*" OR "transspinal stimul*" OR "trans-spinal stimul*" OR "transspinal direct current stimul*" OR "transcutaneous spinal cord stimul*" OR "spinal cord stimul*" OR tsDCS OR tSCS)) | 77 |
| SPORTDiscus with Full Text | EBSCOhost | (TI ("spinal cord injur*" OR "spinal cord lesion*" OR tetraplegi* OR quadriplegi* OR paraplegi*) OR AB ("spinal cord injur*" OR "spinal cord lesion*" OR tetraplegi* OR quadriplegi* OR paraplegi*)) AND (TI ("transcranial magnetic stimul*" OR TMS OR rTMS OR iTBS OR "theta burst stimul*") OR AB ("transcranial magnetic stimul*" OR TMS OR rTMS OR iTBS OR "theta burst stimul*")) AND (TI ("paired associative stimul*" OR "paired associative magnetic stimul*" OR "paired corticospinal-motoneuronal stimul*" OR "paired corticospinal motor neuronal stimul*" OR "paired corticomotoneuronal stimul*" OR "paired stimul*" OR "spinal associative stimul*" OR "Hebbian stimul*" OR "multisite stimul*" OR "multi-site stimul*" OR "dual-site stimul*" OR "dual target stimul*" OR "dual-target stimul*" OR "peripheral nerve stimul*" OR "nerve root stimul*" OR "transspinal stimul*" OR "trans-spinal stimul*" OR "transspinal direct current stimul*" OR "transcutaneous spinal cord stimul*" OR "spinal cord stimul*" OR tsDCS OR tSCS) OR AB ("paired associative stimul*" OR "paired associative magnetic stimul*" OR "paired corticospinal-motoneuronal stimul*" OR "paired corticospinal motor neuronal stimul*" OR "paired corticomotoneuronal stimul*" OR "paired stimul*" OR "spinal associative stimul*" OR "Hebbian stimul*" OR "multisite stimul*" OR "multi-site stimul*" OR "dual-site stimul*" OR "dual target stimul*" OR "dual-target stimul*" OR "peripheral nerve stimul*" OR "nerve root stimul*" OR "transspinal stimul*" OR "trans-spinal stimul*" OR "transspinal direct current stimul*" OR "transcutaneous spinal cord stimul*" OR "spinal cord stimul*" OR tsDCS OR tSCS)) | 93 |
| Total |  |  | 260 |
